# Patient satisfaction and adherence to tuberculosis treatment among adults in the National Tuberculosis Program-Uganda: a cross sectional study

**DOI:** 10.4314/ahs.v26i2.3

**Published:** 2026-06

**Authors:** Harriet Mupere Babikako, Duncan Neuhauser, Achilles Katamba, Kathleen Smyth, Ezekiel Mupere, Christoper Whalen

**Affiliations:** 1 Department of Child Health and Development Center, Makerere University, College of Health Sciences, School of Medicine, Mulago Hill Rd, P.O. Box 7072 Kampala, Uganda; 2 Department of Epidemiology and Biostatistics, Case Western Reserve University, 10900 Euclid Avenue, 44106 Cleveland Ohio, USA; 3 Department of Clinical Epidemiology, Makerere University, College of Health Sciences, School of Medicine, Mulago Hill Rd, P.O. Box 7072 Kampala, Uganda; 4 Department of Pediatrics and Child Health, Makerere University, College of Health Sciences, School of Medicine, Mulago Hill Rd, P.O. Box 7072 Kampala, Uganda; 5 College of Public Health, University of Georgia, 101 Buck Road, Athens, GA 30602

**Keywords:** Patient Satisfaction, Adherence to TB treatment, National Tuberculosis program-Uganda

## Abstract

**Background:**

Non-adherence is a complex challenge to global tuberculosis control because it arises from the interaction of multiple factors, and yet, there are few studies that have evaluated beyond patient factors into the organization and processes of tuberculosis service delivery. We examined the relationship between patient satisfaction and adherence among adult TB patients.

**Methods:**

In a cross-sectional study, we hypothesized that patients with high satisfaction scores were more likely to adhere to prescribed tuberculosis treatment than patients with lower satisfaction scores. 469 new TB cases aged 18 years and older were consecutively recruited from the National TB & Leprosy program-Uganda. The outcome variable was adherence (high was ≥90% Morisky scores) to TB treatment. The exposure variable was patient satisfaction (PS) scores

**Results:**

Of the 469 patients, 29% (135/469) were non adherent). Patients satisfaction scores on the responsiveness to patients' preferences were higher among the adherent compared to the non-adherent, 62.1 ± 8.8 versus 63.8 ± 7.3 (p=0.014), respectively. Patients were very satisfied with the technical quality of care and management of patient's preferences, and were least satisfied with the amount of information about action usage, and effects of tuberculosis medication, regardless of adherence levels. For every unit increase in patient satisfaction scoes for the clinic structure, there was an associated decrease in the level of non-adherence.

**Conclusion:**

Patients' satisfaction with the organization and processes of the clinic was associated with non-adherence to tuberculosis medication. The satisfaction scores for information about action usage, and effects of tuberculosis medication were low.

## Introduction

Despite the development of new classes of TB drugs and Gene X-pert for diagnosis of TB, the tuberculosis epidemic remains a public health threat world-wide. The World Health Organization (WHO) estimated that in 2013 there were 9.0 million new cases of TB disease and 1.5 million deaths from the disease^1^,^2^. An estimated 10.6 million people (95% CI: 9.9–11 million) fell ill with TB in 2021, an increase of 4.5% from 10.1 million (95% CI: 9.5–10.7 million) in 2020; similarly the number of drug resistant TB (450,000) and deaths (1.5 million) from TB appear to be on the rise^3^. In Uganda 90,000 people fell ill with TB and 37.5 TB deaths per 100,000 population in 2021^4^. A major challenge to global tuberculosis control is non-adherence to treatment which is associated with treatment failure, acquired drug resistance, and death^3^,^4^,^5^. In Uganda, it is estimated that up to 11% of tuberculosis patients do not comply with the treatment course at two months^2^; and in Kampala city, where a quarter of the notified cases in Uganda reside, non-compliance is around 20%^8^. Studies have found that promoting compliance through a patient centered approach is more effective than defaulter tracing^3^.

Non-adherence to treatment arises from multiple factors affecting the quality of tuberculosis care^9^, but there are few studies that have evaluated beyond patient factors into the organization and processes of tuberculosis service delivery. In addition, the current tuberculosis program and clinical research have focused only on hard outcome variables such as mortality and microbiologic cure, and have not considered patient preferences such as satisfaction with care, which may be an important determinant in treatment adherence. Further, patients' beliefs and attitudes may influence how patients take drugs^10^.

Patient satisfaction is a legitimate measure of health care quality^11^,^12^ and is defined as a health care recipient's reaction to salient aspects of his or her experience of a service^13^. Satisfaction is assumed to consist of a cognitive evaluation and an emotional reaction to the structure, process and outcome of services. Greater satisfaction may be associated with superior adherence, improved attendance at return visits, and better outcomes, making patient satisfaction, a key determinant, for adherence to treatment^14^,^15^.

Applying the theoretical underpinnings of the Health Belief Model^16^,^17^ such as cues to action and Information-Motivation-Behavioral theory^17^, we can envisage that patients empowered with basic information concerning tuberculosis disease and its treatment, and satisfied with the processes of healthcare service delivery will have better potential to develop behavioral skills of treatment adherence.

In this large cross-sectional study that was conducted in multiple program clinics in urban Uganda, we present results that show the relationship between patient satisfaction and adherence.

## Materials and Methods

### Study design

Using a cross sectional study design, 469 adult (18 or more years of age) patients with confirmed pulmonary tuberculosis were invited to participate. Of the 499 patients screened, thirty patients were excluded, of whom two were too sick to sustain an interview and the rest declined consent for varied reasons. Patients who had completed two, five and eight months of TB treatment were enrolled consecutively between September 2010 and August 2012. Four study sites (Kawempe, Kawala, Komamboga, and Kisenyi) are health centres registering ~30 TB cases per month. The fifth site (Mulago TB Center) is a national TB referral centre registering ~150 cases per month. National tuberculosis program centers are public health centers and services are free to all.

### Measurements

Trained study staff collected socio-demographic data on all participants using standardized forms. Pulmonary tuberculosis was confirmed by sputum smear microscopy. The diagnosis of TB was made by two positive smears for acid fast (AFB) on microscopy or one positive AFB smear with suggestive chest X-ray findings. The Ministry of Health guidelines recommend short course chemotherapy for TB treatment^18^. This is a combination of Rifampicin, Isoniazid, Pyrazinamide, and Ethambutol (RHZE) for the first 2 months then Isoniazid and Ethambutol (EH) given in the continuation phase for 6 months. However, at Mulago teaching hospital, a combination of Rifampicin and Isoniazid (RH) for 4 months in the continuation phase may be prescribed at the discretion of the attending clinician.

Positive enzyme-linked immunosorbent assay for HIV-1 antibodies was diagnosed through routine counseling and testing at the health facility.

Patient satisfaction, the main predisposing factor, was measured using two questionnaires in order to separately tap domains of service satisfaction and satisfaction with information received about tuberculosis medications. The PS 13-item questionnaire^19^ used to assess service satisfaction with three sub scales including a) responsiveness to patients' preferences which deals with satisfaction with the clinic processes, nurses' availability, clinic waiting time and how the patient was treated or handled by staff. The specific questions were documented in earlier articles^19^; b) management of patients' preferences which addresses satisfaction with overall physician care, amount of time spent with the doctor and clinic services in general and c) quality of technical performance which relates to sufficiency of discussion about TB disease, needed treatment, outcomes of treatment, and the associated care and the 17-item Satisfaction with Information about Medicines Scale (SIMS)^20^ have been validated using TB patients in Uganda^19^. The SIMS scale has two sub-scales that assess patients' satisfaction with information about the action and usage of medication (AU) and the potential problems for medication (PPM).

The dependent variable (adherence to tuberculosis medication) was measured by the Morisky scale. Adherence was defined as the Morisky scale cutoff >90% aggregated score. The Morisky psychometric properties and the predictive validity have been described in other studies and found it to have a high sensitivity (90%), moderate specificity (53%) and reliable with Alpha=0.83^21^. The scale was adapted and modified into a six-item scale. The patients were given an exit face-to-face interview by trained study staff. The original Morisky scale^22^ included questions like, “Are you careless at times about taking your medicine?” which was reworded to “Are you always careful about taking your medications?” due to clinician concerns that patients may feel uncomfortable about the word “careless”. Scoring of this item was also reversed to be consistent with the scale properties. Two additional questions were added to scale including *“Do you ever miss taking your medications for any reason”* and *“Do you ever have problems taking your medications”*. For each of the items in the Morisky scale, responses to individual questions were aggregated and scores converted to a 0-to-100 scale, with 100 representing perfect adherence.

The research protocol was reviewed and approved by two institutional review boards (The School of Medicine Research Ethical Committee, Makerere University, and University Hospitals of Cleveland). Written informed consent was obtained from the study participants. The participants were given an information sheet and written consent was obtained. The participants' consent was obtained using the signature block. The person obtaining consent and the principal investigator's signature. Both ethics committees/IRBs approved this consent procedure.

### Statistical analyses

The categorical characteristics were compared using chi-square or Fisher's exact test (where expected counts were less than 5). Responses to individual questions and corresponding subscales for both PS 13-item and SIMS scales were aggregated and scores were converted to a hundred – point scale, with 100 representing the best satisfaction status. The Wilcoxon rank-sum test was used to test differences between groups for all patient satisfaction scores because they were not normally distributed. A series of both univariate and multivariable logistic regression analytic models were fit to test the association between adherence as the dependent variable and patient satisfaction scores as the main independent variable. Other co-variables included HIV status, age (cut off point was the mean age), sex, history of weight loss, level of income, alcohol intake, and patient category (new or transferred in from another clinic), and nutritional status as measured by body mass index<18.5 kg/m^2^. Variables that were associated with adherence/or non-adherence in univariate analysis or were of biological plausibility were included in a series of multivariable logistic regression models. The -2 log likelihood ratio test was used to test differences between the models. Two-way interactions between patient satisfaction scores and the co-variables were evaluated. All analyses were performed using SAS version 9.2 (Cary software, North Carolina SAS Institute Inc. 2004).

## Results

Of the 469 participants who were involved in the analysis, 269 (57%) were young people aged 30 years or less, 155 (33%) participants were HIV positive 7 (1%)) HIV status unknown), HIV status, and a substantial proportion (134 (29%)) of patients were transferred from other centers (Table 1).

**Table 1:** Characteristics of 469 Study Participants Attending the National Tuberculosis Program Centers in Uganda, September 2010-August 2012

Characteristics	Number^a^	%^b^
Age group		
≤ 30 years	269	57
> 30 years	200	43
Sex		
Female	203	43
Male	266	57
TB treatment category		
Completed 2 months	190	40
Completed 5 months	135	29
Completed 6/8months	144	31
Treatment Supporter		
Yes	285	61
No	184	39
HIV status^c^		
Negative	307	66
Positive	155	34
Karnofsky performance score		
> 80	250	53
≤ 80	219	47
Weight loss^d^		
No	382	82
Yes	86	18
Patient category^d^		
New at clinic site	334	71
Transfer in	134	29
Income^d^		
No	126	27
Yes	342	73
Distance^b^		
<5miles	146	31
≥5miles	322	69
Ever smoked		
No	379	81
Yes	90	19
Alcohol Intake		
No	342	73
Yes	127	27
Education		
Primary & below	195	42
Secondary & above	274	58
Employed^c^		
No	125	27
Yes	340	73
Religion		
Protestant	134	28
Catholic	177	38
Others	158	34
BMI (Kg/m^2^)		
>18.5	351	75
≥ 18.5	117	25
Morisky Adherence scale Mean(SD)	92.8(13.1)	
**Patient satisfaction subscales Mean(SD)**		
Responsiveness to patients' preference	63.3(7.8)	
Quality of technical performance	90.3(18.2)	
Management of patients' preferences	92.1(14.9)	
Information about action of medication	49.8(14.9)	
Information about effects of medication	28.2(22.7)	

a Number of patients

b % shows column percent add up to 100%

c HIV status has 7 observations missing

d Patient category, income, and weight loss, each has one observation missing

Of the 469 patients, 29% (135/469) were non adherent (Morisky scores < 90%). The responsiveness to patient's preferences scores were statistically higher among the adherent compared to the non-adherent, 62.1 ± 8.8 versus 63.8 ± 7.3 (p=0.014), respectively (Table 2). Patients were very satisfied with the technical quality of care and the management of patients' preference, and were least satisfied with the amount of information about action usage, and effects of tuberculosis medication regardless of adherence levels (Table 2).

**Table 2:** Patient Satisfaction Scores among 469 study patients by Adherence Status Attending the National Tuberculosis Program, Uganda, September 2010-August 2012

Satisfaction Sub scales	High Adherence	Low Adherence	p-value*
	(N=334)	(N=135)	
	Mean (SD)	Mean (SD)	
Responsiveness to patients' preferences	63.8 (7.3)	62.1 (8.8)	**0.014**
Quality of technical performance	90.9 (17.9)	89.1 (19.0)	0.669
management of patient's preferences	92.6 (13.4)	91.1 (17.3)	0.661
Information about action of medication	49.6 (14.8)	50.3 (15.5)	0.595
Information about effects of medication	28.8 (22.8)	26.6 (22.4)	0.385

* p-value obtained by Wilcoxon rank-sum test; Boldface=significant; SD=standard deviation; >90% Morisky scores=high adherence, <90% Morisky scores=Low adherence

When we examined the association between patient satisfaction scores and adherence status, our data showed a decrease in the odds of non-adherence for every unit increase in the responsiveness to patients' preferences in both univariate and multivariable analysis (Tables 3 and 4, respectively). For example in univariate analysis, a unit increase in responsiveness to patients' preferences was associated with a 3% decrease in the odds of non-adherence (Odds Ratio (OR) 0.974 (95% Confidence Interval (CI): 0.951, 0.998) (Table 4). There were no associations with the rest of other patient satisfaction subscales and non-adherence.

**Table 3:** Satisfaction Sub scales and Associated Odds Ratios for Non Adherence among Adults Tuberculosis Patients at the National Tuberculosis Program Centers, Uganda, 2010-2012: Univariate analysis

Satisfaction Sub scales	Odds Ratio	95% CI
Responsiveness to patients' preferences	0.974	**0.951, 0.998**
Quality of technical performance	0.995	0.984, 1.005
Management of patient's preferences	0.993	0.980, 1.006
Information about action of medication	1.003	0.990, 1.017
Information about effects of medication	0.996	0.987, 1.005

Boldface=significant; CI: Confidence Interval.

**Table 4:** Satisfaction Sub scales, Characteristics and Associated Odds Ratios for Non-Adherence among All TB cases, Adjusting for all the variables in the model, Uganda National Tuberculosis Program, September 2010-August 2012

Satisfaction Sub scales	Overall model
	Odds Ratio (95% CI)^b^
Responsiveness to patients' preferences	0.972 (**0.945, 0.999**)
quality of technical performance	0.997 (0.983, 1.011)
Management of patient's preferences	0.999 (0.980, 1.017)
Information about action of medication	1.009 (0.993, 1.025)
Information about effects of medication	0.997 (0.987, 1.008)
Age group	
> 30 years	-
≤ 30 years	1.008 (0.678, 1.632)
Sex	
Female	-
Male	0.714 (0.466, 1.094)
Patient category	
New patient at clinic	-
Transfer in	0.649 (0.399, 1.056)
Karnofsky score	
> 80	-
≤ 80	1.132 (0.874, 1.729)
Weight loss	
No	-
Yes	0.874 (0.505, 1.514)
Alcohol intake	
No	-
Yes	1.695 (**1.069, 2.689**)
Level of income	
Some	-
None at all	0.702 (0.416, 1.184)
HIV status	
Negative	-
Positive	1.02 (0.64, 1.65)

Abbreviations: CI, confidence interval; Boldface=significant

a ORs and 95% CI from stratified logistic regression models, adjusted for age, sex, patient category, karnofsky score, weight loss, alcohol intake, income.

b Odds Ratios and 95% CIs from overall logistic regression models, adjusted for age, sex, patient category, karnofsky score, weight loss, alcohol intake, income; Overall model has both HIV positive and Negative patients.

### Satisfaction and HIV

The satisfaction scores were in general higher among 155 HIV positive patients compared to 307 HIV negative (Table 5). Patients had very high satisfaction scores for technical quality and hospital care and very low satisfaction scores for information about action and usage, and about effects of the tuberculosis medication regardless of HIV status (Table 6). Significant differences in patient satisfaction scores between HIV positive and negative patients were found. These included: satisfaction with the responsiveness to patients' preferences, 64.5 ± 6.1 versus 62.6 ± 6.1; satisfaction with the management of patients' preference, 93.4 ± 14.3 versus 91.4 ± 14.8; and satisfaction with information on effects of the drug, 32.0 ± 23.8 versus 26.4 ± 22.0, respectively (Table 5).

**Table 5:** Satisfaction Sub scales, Mean Scores among 469 study Patients by HIV Status Attending National Tuberculosis Program Centers, Uganda, September 2010-August 2012

Satisfaction subscales	HIV Negative (N=307)	HIV Positive (N=155)	p-value*
	Mean (SD)	Mean (SD)	
General clinic setting	62.6 (8.6)	64.5 (6.1)	**0.016**
Technical quality	90.1 (17.3)	90.9 (19.9)	0.054
Hospital care	91.4 (14.8)	93.4 (14.3)	**0.023**
Information about action of medication	49.7 (15.8)	50.6 (13.3)	0.749
Information about effects of medication	26.4 (22.0)	32.0 (23.8)	**0.023**

* p-value obtained by Wilcoxon rank-sum test; Boldface=significant; SD=standard deviation; 7 observation of HIV have unknown status

**Table 6:** Satisfaction Sub scales, Mean Scores among 469 study Patients by HIV Status Attending National Tuberculosis Program Centers, Uganda, September 2010-August 2012

Satisfaction Sub scales	Overall model	HIV negative	HIV positive
	Odds Ratio (95% CI)^b^	Odds Ratio (95% CI)^a^	Odds Ratio (95% CI)^a^
Responsiveness to patients' preferences	0.972 (**0.945, 0.999**)	0.964 (**0.933, 0.995**)	1.012 (0.940, 1.088)
quality of technical performance	0.997 (0.983, 1.011)	1.005 (0.986, 1.024)	0.984 (0.961, 1.007)
Management of patients' preferences	0.999 (0.980, 1.017)	0.989 (0.966, 1.011)	1.016 (0.981, 1.053)
Information about action of medication	1.009 (0.993, 1.025)	1.006 (0.986, 1.026)	1.023 (0.991, 1.056)
Information about effects of medication	0.997 (0.987, 1.008)	0.998 (0.984, 1.012)	0.992 (0.975, 1.010)
Age group			
> 30 years	-	-	-
≤ 30 years	1.008 (0.678, 1.632)	1.128 (0.618, 2.059)	0.913 (0.424, 1.967)
Sex			
Female	-	-	-
Male	0.714 (0.466, 1.094)	0.683 (0.391, 1.192)	0.608 (0.288, 1.284)
Patient category			
New patient at clinic	-	-	-
Transfer in	0.649 (0.399, 1.056)	0.513 (**0.277, 0.950**)	1.115 (0.463, 2.688)
Karnofsky score			
> 80	-	-	-
≤ 80	1.132 (0.874, 1.729)	1.285 (0.751, 2.196)	0.932 (0.434, 1.999)
Weight loss			
No	-	-	-
Yes	0.874 (0.505, 1.514)	1.086 (0.514, 2.295)	0.667 (0.278, 1.597)
Alcohol intake			
No	-	-	-
Yes	1.695 (**1.069, 2.689**)	1.972 (**1.091, 3.564**)	1.180 (0.527, 2.639)
Level of income			
Some	-	-	-
None at all	0.702 (0.416, 1.184)	0.722 (0.383, 1.361)	0.481 (0.160, 1.449)

Abbreviations: CI, confidence interval; Boldface=significant

a ORs and 95% CI from stratified logistic regression models, adjusted for age, sex, patient category, karnofsky score, weight loss, alcohol intake, income.

b Odds Ratios and 95% CIs from overall logistic regression models, adjusted for age, sex, patient category, karnofsky score, weight loss, alcohol intake, income; Overall model has both HIV positive and Negative patients.

We found a significant interaction between HIV status and patient satisfaction score for responsiveness to patients' preferences on adherence (OR = 1.072; (95% CI: 1.000, 1.150) (p=0.049). We therefore performed stratified multivariable logistic regression analysis according to HIV status. In stratified multivariable analysis (Table 6),

*“Odds ratios among HIV negative patients showed an increased level of adherence per unit change in satisfaction score for responsiveness to patient's preferences ((OR = 0.964; (95% CI: 0.933, 0.995)) when all other factors in the model were held constant.*

There were minimal changes in level of non-adherence among HIV positive patients with increase in level of satisfaction scores for responsiveness to patient's preferences. Adherence increased among 49% of HIV negative patients who transferred in from other clinics compared to new clinic patients, while holding other variables in the model constant (OR = 0.513 [95% CI: 0.277, 0.950]).

Among HIV negative patients, given other variables in the model are constant, patients with a history of alcohol intake were 97% (OR = 1.972 (95% CI: 1.091, 3.564) more likely to be non-adherent compared to those who did not ingest alcohol (Table 6).

## Discussion

The main aim of the study was to compare patient adherence as measured by a series of self-report questions (Morisky scale) among patients at 2, 5, and 8 months of treatment for tuberculosis (TB) disease with levels of self-reported patient satisfaction with healthcare received. Secondary outcomes included a limited number of patient characteristics that may affect adherence. While the findings are not strong and many other factors relating to non-adherence were not significant, the data provide insight into adherence in a population with a high incidence of TB, and the main message of the study is an important reminder that TB treatment requires more than just a willing patient in order to succeed.

Perhaps the larger concern to highlight is the very low satisfaction with information on action and effects of medication. In my experience, it is here where we stand to gain the most because adherence is difficult to maintain if one does not recognise the difference between a common nuisance side effect (that may even be corrected with changes in dose or how and when medication is taken) and a rare life threatening complication.

In this cross-sectional study, 469 adult patients with pulmonary tuberculosis were recruited from five national TB and Leprosy (NTLP) program clinics in Uganda. Using the Morisky scale, our data revealed that 29% of the participants were non adheret, and poor satisfaction scores were more likely to be associated with poor adherence. Patients' satisfaction with responsiveness to patients' preference was associated with significant reduction in non-adherence to tuberculosis medication. HIV negative patients had significant reduction in non-adherence with a unit increase in satisfaction scores for responsiveness to patients' preference; there was minimal effect among HIV positive patients with a unit increase in satisfaction scores for health facility structure. The satisfaction scores for information about action, usage, and effects of tuberculosis medication were markedly sub-optimal. There were minimal changes in levels of adherence as regards changes in satisfaction scores for technical quality of care; hospital care; information about action and usage of, and about effects of tuberculosis medication.

The findings in this study suggest that the organization of the clinic and information about action and usage of, and about effects of tuberculosis medication may be important determinants contributing to non-adherence. Previous studies have explored satisfaction with service care among tuberculosis patients and development of satisfaction tools; and there is dearth of information as regards patient satisfaction in tuberculosis programs^19^.

TB programmes have changed dramatically in the Uganda and sub-Saharan African context in the past decade. A national TB strategic plan was launched in 2020 where one of the strategic objective described to increase treatment success rate from 64% to 90% through increase of patient education including availing education materials, health worker and client involvement in patient education^21^. However, there are concerns about sources to finance this strategic plan hence the lack of the clinic environment or Standard Operatng Procedures for reviewing medication with clinic clients.

The organization and processes of health care in Uganda and other countries in sub-Saharan Africa have been reported to be inefficient in diagnosis and delivery of tuberculosis treatment^23^. This could be one possible explanation for our study findings because structure and process are key determinants of satisfaction^13^,^24^,^25^. Patients' experiences with the responsiveness to patients' preferences in tuberculosis unit appears to be a major hindrance to adherence particularly with HIV negative patients who may not get additional supports beyond tuberculosis medication such as counseling and sometimes food rations offered to HIV positive patients. Patients' experiences in receiving care and communication with care providers^26^ is another explanation; and in line with the Information-Motivation-Behavioral theory^17^, empowered patients with adequate information about tuberculosis medication are likely to develop appropriate behavioral skills that promote adherence. Patients in our study had markedly suboptimal satisfaction scores for information about tuberculosis medication to have developed behavioral skills or to have reached a threshold that could promote behavior change to impact significantly on adherence.

Our study findings have important implications. Tuberculosis programs and clinicians need to pay attention to the organization and responsiveness to patients' preferences and processes of tuberculosis clinics and information about tuberculosis medication offered to patients as key factors in order to deliver patient-centered healthcare to achieve adherence to prescribed tuberculosis treatment^27^,^28^; just as it may be important to maintain satisfaction to technical quality of and hospital care. The findings in this study identify short comings in the current Ugandan tuberculosis program for achieving standard 9 of the International Standards for Tuberculosis Care. Standard 9 requires that tuberculosis programs foster and assess adherence utilizing a patient-centered approach to treatment administration based on the patient's needs and mutual respect between patient and provider^27^,^28^.

Although our findings reflect important suggestions for tuberculosis programs, the interpretations are not without limitations.

There are a lot of variables considered, and with an alpha of 0.05, one could expect to find a spurious result for every twenty comparisons. The authors should therefore be cautious with regard to reporting the main findings. While a satisfaction score of 63.8 and 62.1 on the Morisky scale may be statistically significant, a minor change with a confidence interval so close to 1 would not be likely motivate a clinician to improve clinic responsiveness to patients' preferences to improve adherence.

Self-reported adherence in response to questions such as “Are you always careful about taking your medications?” may not yield robust adherence categories however, there are no gold standard tools that can measure adherence. Besides, other adherence measures are not without limitations.

We used a cross-sectional design therefore the associations may not be causal.

In addition, caution with regard to the findings related to alcohol, as intake appears to be measured by ever/never (based upon Table 1). We report that those who use any alcohol are 97% more likely to be non-adherent to TB treatment. While alcohol dependence certainly affects a patients' ability to adhere, this finding is likely an underestimate of those with alcohol dependence and an overestimate of those who drink occasionally. Smoking and alcohol are best measured by a combination of current use and amount to better articulate levels that would be of clinical concern which was not done in this study. However, we took care to recruit participants at varying stages of tuberculosis treatment (2, 5, and 8 months) and recruited a large number of participants from multiple clinics in an urban setting to ensure a varied sample. The study staff were independent tuberculosis service provision.

## Conclusion

Patients' satisfaction with responsiveness to patients' preferences and processes of the clinic was associated with non-adherence to tuberculosis medication. Patients with low levels of adherence were associated with poor satisfaction scores. HIV positive patients had better satisfaction scores compared to HIV negative individuals. The satisfaction scores for information about action usage, and effects of tuberculosis medication were markedly sub-optimal. Patients' satisfaction with the technical quality of care; management of patients' preference; information about action and usage of, and about effects of tuberculosis medication was not related to adherence. Further studies are required about the current TB strategic plan, 2020-2025 and the related TB outcomes including patient preferences, clinic and organization processes in relation to adherence to TB treatment in our context in order to develop more specific guide for improvement since information about tuberculosis medication given to patients as key factors that help to deliver patient-centered healthcare and hence desired levels of adherence to prescribed tuberculosis treatment.
